# Accelerating the *Mdx* Heart Histo-Pathology through Physical Exercise

**DOI:** 10.3390/life11070706

**Published:** 2021-07-17

**Authors:** Jacopo Morroni, Leonardo Schirone, Daniele Vecchio, Carmine Nicoletti, Luca D’Ambrosio, Valentina Valenti, Sebastiano Sciarretta, Biliana Lozanoska-Ochser, Marina Bouchè

**Affiliations:** 1 Department of Anatomical, Histological, Forensic Medicine and Orthopedic Sciences, Section of Histology and Embryology, Sapienza University of Rome, 00161 Rome, Italy; jacopo.morroni@uniroma1.it (J.M.); carmine.nicoletti@uniroma1.it (C.N.); biliana.lozanoska-ochser@uniroma1.it (B.L.-O.); 2 Department of Medical and Surgical Sciences and Biotechnologies, Sapienza University of Rome, 00161 Rome, Italy; leonardo.schirone@uniroma1.it (L.S.); daniele.vecchio@uniroma1.it (D.V.); L.dambrosio@uniroma1.it (L.D.); sebastiano.sciarretta@uniroma1.it (S.S.); 3 Department of Cardiology, Ospedale Santa Maria Goretti, 04100 Latina, Italy; valevale2012@hotmail.com; 4 IRCCS Neuromed, Via Atinense 18, 86077 Pozzilli, Italy

**Keywords:** DMD, *mdx*, exercise, fibrosis, heart, animal model

## Abstract

Chronic cardiac muscle inflammation and fibrosis are key features of Duchenne Muscular Dystrophy (DMD). Around 90% of 18-year-old patients already show signs of DMD-related cardiomyopathy, and cardiac failure is rising as the main cause of death among DMD patients. The evaluation of novel therapies for the treatment of dystrophic heart problems depends on the availability of animal models that closely mirror the human pathology. The widely used DMD animal model, the *mdx* mouse, presents a milder cardiac pathology compared to humans, with a late onset, which precludes large-scale and reliable studies. In this study, we used an exercise protocol to accelerate and worsen the cardiac pathology in *mdx* mice. The mice were subjected to a 1 h-long running session on a treadmill, at moderate speed, twice a week for 8 weeks. We demonstrate that subjecting young *mdx* mice (4-week-old) to “endurance” exercise accelerates heart pathology progression, as shown by early fibrosis deposition, increases necrosis and inflammation, and reduces heart function compared to controls. We believe that our *exercised mdx* model represents an easily reproducible and useful tool to study the molecular and cellular networks involved in dystrophic heart alterations, as well as to evaluate novel therapeutic strategies aimed at ameliorating dystrophic heart pathology.

## 1. Introduction

DMD is an X-linked genetic disease caused by mutations in the dystrophin gene. The lack of functional dystrophin destabilizes the muscle sarcolemma, causing progressive muscle damage [1,2,3]. Chronic inflammation in both skeletal and cardiac muscle leads to fibrotic tissue deposition that impairs muscle function [1,2,3,4]. It is estimated that almost 60% of 10-year-old boys present clear signs of cardiomyopathy, and this percentage increases up to 90% among 18-year-old patients [4,5,6]. Along with respiratory failure, cardiac failure represents the main cause of death in DMD patients [1,3,4].

At present, there is no cure for DMD, and the only recommended treatment is the use of corticosteroids [1,3,4], supporting the notion that the inflammatory compartment plays an important role in disease progression. Corticosteroid treatment slows down DMD progression in skeletal muscle, delaying the loss of ambulation and the need for assisted ventilation by improving muscle strength and dampening inflammation [1,3]. However, long-term use of corticosteroids is associated with severe adverse effects, including bacterial and viral infections, as well as metabolic disorders. Although corticosteroids are believed to be effective in delaying cardiomyopathy onset in DMD patients, there is some controversy regarding the reliability of several large-scale studies [4,5]. There is therefore an urgent need for novel therapeutic approaches for the treatment of cardiomyopathy in DMD. In order to evaluate the effectiveness of novel therapeutic approaches, it is essential to have at our disposal appropriate animal models that genetically and phenotypically mirror the human pathology. The widely used DMD animal model, the *mdx* mouse, resembles the human pathology in terms of the genetic mutation and early skeletal muscle phenotype [7,8,9]. However, the disease progression is quite different in comparison to human patients [9]. For example, the lifespan of *mdx* mice is only 20% lower than that of wild-type mice [10], while human patients, without assisted ventilation, die before they reach 25 years of age [11,12]. Moreover, *mdx* mice present a milder cardiac pathology compared to human patients, with a slower onset and strong individual variation [7,8,9,10]. The absence of an animal model that closely resembles the severity of DMD cardiomyopathy has hindered large-scale and reliable studies of potential therapeutic strategies aimed at ameliorating the dystrophic heart phenotype. Previous studies have described different mouse models displaying worsened heart pathology that were generated by additional genetic manipulations [8], such as deletion of utrophin in the double mutant *mdx*/utrn−/− [13,14], which was recently further manipulated to resemble human heart pathology in the Fiona/dko mouse model [15]. Another strategy was to eliminate or humanize the telomerase gene in *mdx* mice, as in the *mdx/mTR* model [16].

In this study, we aimed to accelerate and worsen the heart pathology in *mdx* mice, through exercise, avoiding additional genetic modifications. Unlike voluntary exercise, which has been found by some [17,18,19] but not all studies [20,21] to be beneficial, we reasoned that forced exercise might accelerate the *mdx* heart pathology. Indeed, “forced” running exercise on either a treadmill or by swimming is largely employed as a protocol to worsen the *mdx* phenotype and/or to evaluate the efficacy of therapeutic interventions with pharmacotherapies [22,23,24,25,26,27]. However, most of the studies have been conducted to evaluate worsening in skeletal muscle phenotype, while only a few of them have evaluated effectiveness in accelerating the heart pathology in young *mdx* mice [28,29,30,31,32,33,34]. Exercised *mdx* hearts are characterized by increased transcription of pro-fibrotic factors such as TGFβ [31], activation of p38 MAPK, JNK1, ERK1/2, or calcineurin signaling [29], and increased cardiac fibrosis [32] or worsened heart function [34]. However, a detailed investigation of the dystrophic *mdx* heart phenotype following exercise, including histo-pathological and functional features, as well as inflammation, is lacking.

Taking into account the protocols described previously, we hypothesized that moderate but long-lasting physical exercise might be the most effective approach for worsening heart phenotype and function in *mdx* mice, avoiding extensive damage to skeletal muscle. Here, we demonstrate that treadmill long-term “endurance” exercise protocol accelerates *mdx* heart fibrosis, increases inflammation, and reduces *mdx* heart function. To the best of our knowledge, ours is the first study to investigate the effect of exercise on cardiac immune infiltration and ventricular fibrosis, both of which are key features of human DMD-related cardiomyopathy. Based on the histo-pathological features observed, this protocol could be a useful tool to study DMD-related cardiomyopathy treatments.

## 2. Materials and Methods

Mice: C57BL/10ScSn-*Dmd^mdx^* mice were purchased from the Jackson Laboratory (Bar Harbor, ME, USA). The mice were housed in the Histology Department-accredited animal facility at the University of Sapienza. All the procedures were approved by the Italian Ministry for Health and were conducted according to the EU regulations and the Italian Law on Animal Research. Only males were used.

Histology: Heart ventricles were embedded in tissue-freezing medium after dissection (O.C.T. Compound, Sakura, 4583) and snap-frozen in liquid nitrogen-cooled isopentane. Frozen heart ventricles were cut into 8 μm sections and stored at −20 °C until use. Histochemistry and immunofluorescence analyses were performed as previously described [35,36]. Briefly, for histological analysis, the sections were stained with hematoxylin/eosin or with Sirius red/picric acid (both from Sigma-Aldrich, St. Louis, MO, USA). Quantification of collagen deposition was determined using the Color Deconvolution plugin (by G. Landini) of ImageJ software. For immunostaining, permeabilization in methanol (6 min at −20 °C) was performed on cryosections after fixation. Sections were incubated with anti-mouse IgG antibody coupled to Alexa Fluor 488 (Life Technologies, Carlsbad, CA, USA). Nuclei were counterstained with Hoechst 33,342 (Fluka, Charlotte, NC, USA). The sections were photographed in a Zeiss Axioskop 2 Plusfluorescence Microscope and the images were analyzed using Image J software.

Flow Cytometry: Cytofluorimetric analysis was performed as previously described [37,38]. Briefly, hearts were collected and cut into small pieces with a blade, and then incubated with collagenase type IV for 1 h and 30′ at 37 °C with agitation. The obtained cell suspension was passed through a 70 μm and then a 40 μm cell strainer; the cells were then counted on a hemocytometer, collected by centrifugation at 1200 rpm, and suspended in 200 μL of calcium/magnesium-free PBS (phosphate buffered saline) with 2% FBS (foetal bovine serum). They were then divided in two tubes for the staining. The cells were incubated on ice for 30 min with the following antibodies: CD45 PE/Cy7, F4/80 APC, Ly6g PE Fluor 610, CD11b APC/Cy7, CD206 PERCP/Cy5.5 Ly6c BV-510, I-Ab FITC (tube 1) and CD45 PE/Cy7, CD3 PERCP Cy5.5, B220 BV-510, CD4 AF488, and CD8 PE (tube 2), all by Biolegend, San Diego, CA, USA). Cells were then washed with 3 mL of calcium/magnesium-free PBS and resuspended in 200 μL of calcium/magnesium-free PBS with 2% FBS. Samples were acquired with a CyAn ADP (Agilent DAKO, Santa Clara, CA, USA), and acquired data were analyzed using FlowJo software version 10.

qRT-PCR: Total RNA from muscle tissues was extracted using the TRIsure solution (Bioline, London, UK) and converted in cDNA using the SensiFast cDNA Synthesis kit from Bioline, according to supplier’s instructions. PCR amplification was performed using the SensiMix SYBR Lo-Rox Mix, from Bioline, following the manufacturer’s protocol. All PCR reactions were carried out in duplicate. All qPCR results are expressed as relative ratios of the target cDNA transcripts to GAPDH and normalized to those of the reference condition. To amplify the genes of interest, we used the following primers pairs: GAPDH (for) 5′-ACCCAGAAGACTGTGGATGG-3′ (rev) 5′-CACATTGGGGGTAGGAACAC-3′: Collagen1α (for) 5′-ACCCAGAAGACTGTGGATGG-3′ (rev) 5′-CAGATTGGGGGTAGGAACAC-3′, CTGF (for) 5′-AGAACTGTGTACGGAGCGTG-3′ (rev) 5′-AGAACTGTGTACGGAGCGTG-3′; TGFβ (for) 5′-CCCGAAGCGGACTACTATGC-3 (rev) 5′-CATAGATGGCGTTGTTGCGG-3′.

Treadmill Exercise: The exercise was carried out using a five-lane motorized treadmill (LE 8710, PanLab S.L.U., Barcelona, Spain) supplied with shocker plates. To set up the exercise protocol, we followed the general indication contained in the S.O.P. DMD_M_2.1.001 by De Luca et al., 2008. After 10 min of acclimation on the stationary treadmill, the exercise started with 10 min-long «warm-up» session, in order not to stress or exhaust the mice too fast. Warm-up started at a speed of 10 cm/s, with an increase of 2 cm/s every 2 min. Then, 50 min of exercise was performed at a constant speed of 20 cm/s. We recommend spacing out two sessions of 25 min of exercise (20 cm/s) with a 5 min-long pause. An important adjustment we followed was not to use electric shocker plates to induce the mice to the run, instead using gentle manipulation or a physical obstacle. This adjustment reduced stress in mice and sensibly increased their ability to complete the exercise session, avoiding exhaustion.

Echocardiography: Echocardiographic analyses were performed as previously described [39,40]. Mice were anesthetized with 2.5% avertin (Sigma, St. Louis, MO, USA, T48402) to perform echocardiographic functional analyses of the fractional shortening of the left ventricle. Echocardiography was performed in M mode using a VEVO 3100 (Visualsonics, Toronto, ON, Canada) with a mx550d probe.

Statistical Analysis: All statistical analyses were performed using Prism software, version 6 (GraphPad Software, Inc., La Jolla, CA, USA). Data are presented as mean ± SEM. Unpaired two-tailed Student’s *t*-test was used for statistical comparison between two groups, and one-way ANOVA (with Bonferroni correction for multiple comparisons) was used for comparisons between multiple groups. A *p* value of ≤ 0.05 was considered statistically significant.

## 3. Results

### 3.1. Mdx Mice Display Late Onset Heart Fibrosis

**Figure 1:**
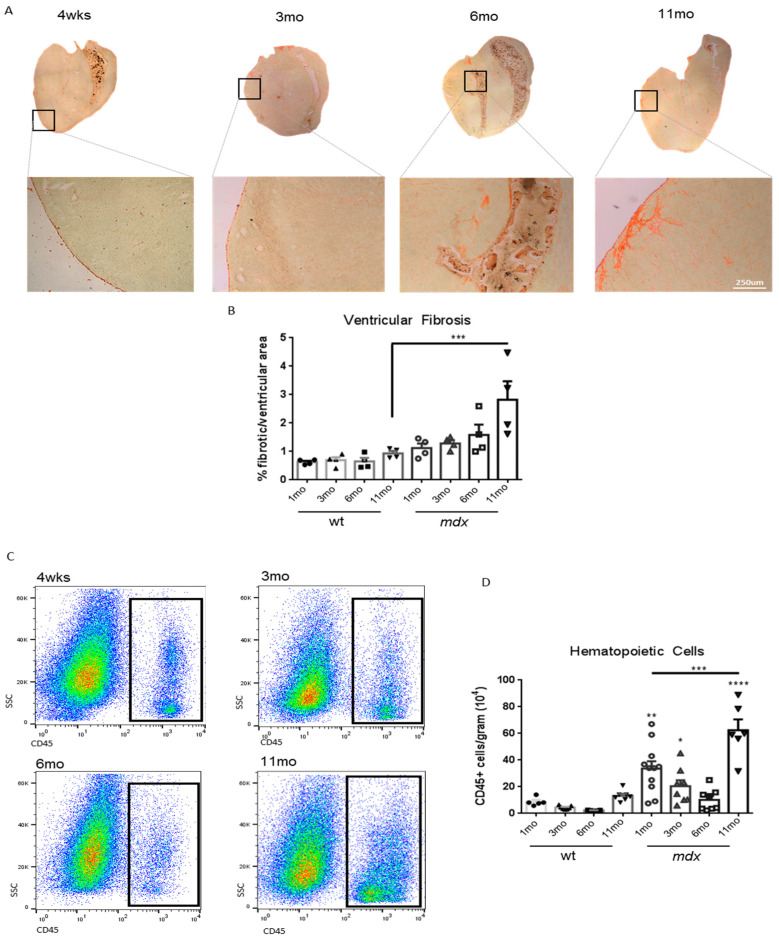
Kinetics of fibrosis establishment and immune infiltration in *mdx* hearts: (**A**) representative images of Sirius red collagen staining of heart ventricles cryosections from *mdx* mice of 1, 3, 6, and 11 months of age. (**B**) Quantification of ventricular fibrotic area, determined as in (**A**), expressed as percentage over total area, calculated with ImageJ Color Deconvolution plugin. *n* = 4 independent samples. The level of ventricular fibrotic area determined in age-matched wild-type mice, as in (**A**), is also shown. (**C**) Representative images of cytofluorimetric analysis of cells derived from *mdx* mice hearts of 1, 3, 6, and 11 months of age, showing the gating for CD45+ cells, as determined after excluding debris, doublets, and dead cells. (**D**) Quantification of the CD45+ cells infiltrating wild-type or *mdx* heart at the indicated ages, expressed as number per gram of tissue. *n* = 5–11 independent samples. Data are shown as mean ± S.E.M; * = *p* < 0.05, ** = *p* > 0.01, *** = *p* < 0.001, **** = *p* < 0.0001, ordinary one-way ANOVA with Bonferroni correction for multiple comparisons.

To examine the kinetics and features of dystrophic heart in *mdx* mice, we analyzed the progression of ventricular fibrosis in 1-, 3-, 6-, and 11-month-old *mdx* mice, compared to age-matched wild-type mice. The extent of ventricular fibrosis was evaluated by Sirius red staining of heart cryosections and analyzed using ImageJ software. As expected, heart fibrosis starts to become evident at around 11 months of age, which is quite late, considering that the average lifespan of a mouse is 26/28 months [10] (Figure 1A,B). Since fibrosis might result from previous necrosis and sustained inflammation [37,38], we analyzed the kinetics of immune cells infiltrating the *mdx* heart. Our gating strategy is shown in Supplementary Figure S1. Non-muscular mononuclear cells were isolated from hearts derived from *mdx* mice at 1, 3, 6, and 11 months of age, as above. Hematopoietic-infiltrating cells were identified by flow cytometry as CD45+ cells. Interestingly, we found that immune cells infiltrated the *mdx* heart in two distinct waves; a first one around 1 month of age and the second around 11 months of age, when fibrosis is established (Figure 1C,D). Both myeloid cells (CD45+/CD11b+ cells) and T cells (CD45+/CD3+ cells) followed the same two-wave kinetics (Supplementary Figure S2).

### 3.2. The Exercise Protocol Accelerates Fibrosis Onset in Mdx Heart

**Figure 2:**
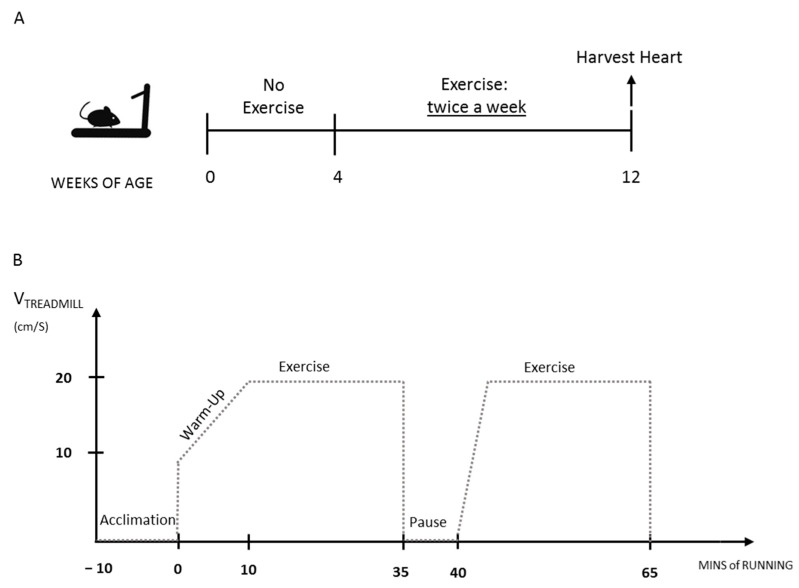
Description of the exercise protocol: (**A**) timeline of the exercise protocol: 4-week-old mice were exercised twice a week for 2 months. (**B**) Description of the exercise session: mice were exercised on the treadmill with no inclination. Each session consists in 1 h of running at moderate speed (max 20 cm/s). After 10 min of acclimation on the stationary treadmill, the exercise starts with 10 min-long «warm-up» session, when the speed is slowly increased from 10 cm/s to 20 cm/s. Two session of exercise at a constant speed of 20 cm/s are spaced out by a 5 min-long pause. Instead of shocker plates, gentle manipulation or physical obstacles are used to force running.

In order to generate a reliable mouse model to study DMD-related cardiomyopathy, we tested the efficacy of physical exercise in accelerating *mdx* heart pathology. Following the established SOPs included in the TREAT-NMD [41], we designed a moderate, long-lasting exercise protocol, as described in Figure 2. Briefly, 4-week-old mice were subjected to a 1 h-long running session on a treadmill at a moderate speed, twice a week for 8 weeks, up to three months of age.

**Figure 3:**
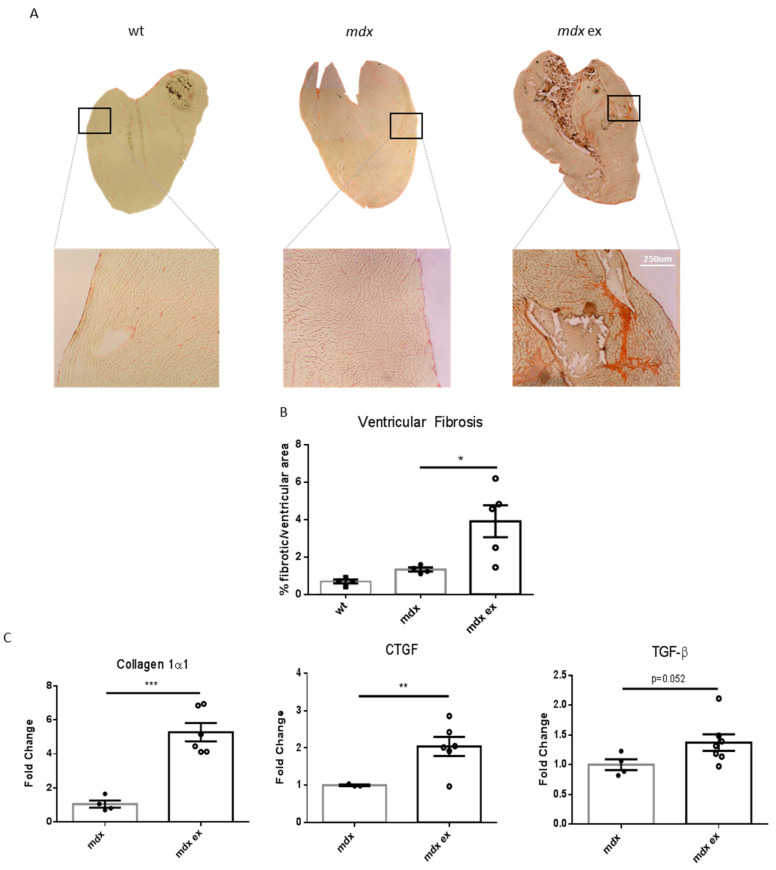
Increased fibrotic tissue deposition in exercised *mdx* heart ventricles: (**A**) representative images of Sirius red collagen staining of heart ventricles cryosections from wild-type C57/BL10, exercised *mdx*, and control *mdx* mice. (**B**) Quantification of heart ventricular fibrosis, determined by Sirius red collagen staining as in (**A**). Data are expressed as percentage of ventricular fibrotic area over total ventricular area, as determined with ImageJ Color Deconvolution plugin by G. Landini. *n* = 4–5 independent samples/group; * = *p* < 0.05, ordinary one-way ANOVA with Bonferroni correction for multiple comparisons. (**C**) qRT-PCR on total heart RNA in exercised and control *mdx* for the pro-fibrotic factors collagen1α1, connective tissue growth factor (CTGF), and TGF-β. Data expressed as fold change (2^-ddCT^) normalized with GAPDH. Data are shown as media ± S.E.M. *n* = 4, 6 independent samples; * = *p* < 0.05, ** = *p* < 0.01, *** = *p* < 0.001, unpaired *t*-test w/Welch’s correction.

To examine the effect of our exercise protocol on heart fibrosis, cryosections of the heart ventricles were stained with Sirius red, and the extent of collagen deposition was determined using ImageJ software. As shown in Figure 3, no fibrotic tissue was observed within the heart of wild-type or 3-month-old *mdx* mice, used as controls, whereas ventricular fibrosis was evident in the heart of exercised *mdx* mice. Interestingly, the percentage of fibrotic ventricular area in exercised *mdx* mice was very similar to that observed in older (11-month-old) unexercised *mdx* (Supplementary Figure S3). In agreement with the histological data, qRT-PCR showed that the level of expression of both collagen 1α1 and connective tissue growth factor (CTGF) mRNA, associated with cardiac fibrosis development [42], was significantly higher in the heart of exercised *mdx* mice compared to controls, together with a slight increase in TGF-β transcript (Figure 3C). Interestingly, tibialis anterior fibrosis was only moderately increased by the exercise protocol, and the diaphragm showed almost no change when comparing unexercised and exercised *mdx* (Supplementary Figure S4). Overall, these results demonstrate that endurance exercise accelerates fibrotic tissue deposition in the dystrophic heart.

### 3.3. The Exercised Mdx Mice Display Increased Cardiomyocyte Necrosis and Cell Infiltration

**Figure 4:**
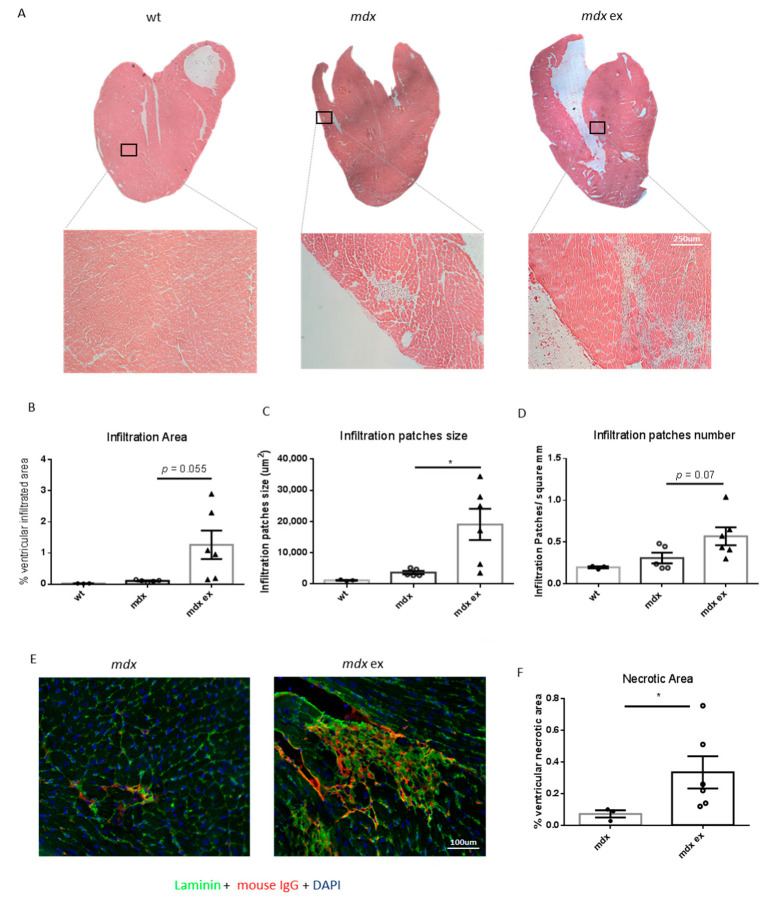
Increased non-muscle cells’ infiltration area and cardiomyocyte necrosis in exercised *mdx* heart ventricles: (**A**) representative H&E staining of ventricular cryosections derived from wild-type, exercised, and control *mdx*. (**B**) Quantification of cell infiltration area expressed as percentage over total ventricular area, as determined in H&E stained cryosections. (**C**) Quantification of the size of cell infiltration patches, expressed in µm^2^. (**D**) Quantification of the number of cell infiltration patches, normalized per mm^2^. Data from B, C, and D are shown as mean ± S.E.M., *n* = 3–6 independent samples/group; * = *p* < 0.05, ordinary one-way ANOVA with Bonferroni correction for multiple comparisons. All quantifications were calculated using ImageJ software. (**E**) Representative images of immunofluorescence of ventricular cryosection from exercised and control *mdx* using an anti-mouse IgG antibody staining. (**F**) Quantification of ventricular necrotic area expressed as percentage over total ventricular area, calculated using ImageJ software. Data are shown as mean ± S.E.M *n* = 3/6 independent samples * = *p* < 0.05, unpaired *t*-test w/Welch’s correction.

To assess cardiac muscle organization, heart ventricles cryosections were stained with hematoxylin and eosin (H&E). We found no obvious alterations in cardiac muscle tissue organization in exercised *mdx* compared with age-matched wild-type or *mdx* mice; however, a slight increase in cell infiltration area was observed, although this increase did not reach statistical significance (Figure 4A,B). Interestingly, cell infiltration patches were both higher in number and bigger in size in the heart of exercised *mdx* mice compared to unexercised controls (Figure 4C,D). Immune cell infiltration of the dystrophic heart is often triggered by the necrotic cell death of cardiomyocytes [43], and fibrotic tissue deposition usually follows muscle cell death in both dystrophic skeletal and cardiac muscle [44]. We therefore analyzed cardiomyocyte necrosis after the exercise protocol. Interestingly, there was a significant increase in the ventricular necrotic area in exercised *mdx* heart compared to unexercised controls, as shown by immunofluorescence analysis using an anti-mouse IgG antibody (Figure 4E,F). Taken together, these results demonstrate that the exercise protocol worsens the dystrophic heart phenotype in terms of increased cell infiltration and cardiomyocytes necrosis.

### 3.4. Inflammatory Cell Infiltration Is Increased in the Exercised Mdx Heart

**Figure 5:**
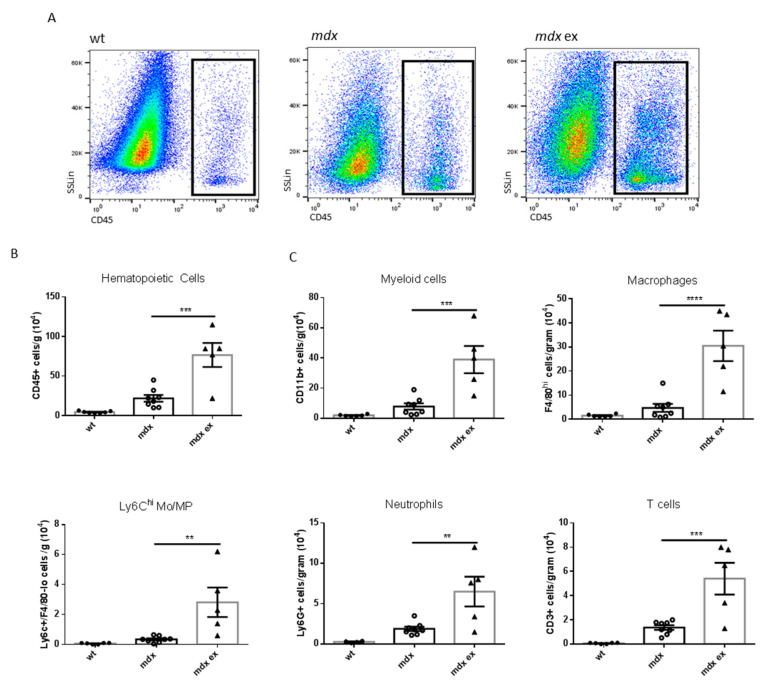
Cytofluorimetric analysis of the immune population isolated from wild-type, exercised *mdx*, and control *mdx* hearts: (**A**) representative images showing the gating for CD45+ infiltrating cells in wild-type exercised and control *mdx* hearts, after excluding debris, doublets, and dead cells. (**B**) Quantification of total hematopoietic cells infiltrating wild-type, exercised *mdx*, and control *mdx* hearts, identified as CD45 positive and expressed as number of cells normalized per gram of tissue. (**C**) Quantification of the immune subpopulation infiltrating the heart of wild-type, exercised *mdx*, and control *mdx* mice, expressed as number of cells normalized per gram of tissue. The cell populations were identified as indicated: total myeloid cells as CD11b+, macrophages as Ly6c-/F4/80^hi^, freshly recruited monocytes as Ly6c^hi^/F4/80-, neutrophils as Ly6g+, and T cells as CD3+. Data are shown as mean ± S.E.M., *n* = 6/8/5 independent samples. ** = *p* < 0.01, *** = *p* < 0.01, **** = *p* < 0.0001, ordinary one-way ANOVA with Bonferroni correction for multiple comparisons.

Most fibrotic disorders are characterized by persistent inflammation, leading to the production of growth factors, proteolytic enzymes, and pro-fibrotic cytokines, which stimulate the deposition of fibrotic tissue [43,44]. Since we observed an increase in heart fibrosis, necrosis, and ventricular infiltration area, we then analyzed the level and the type of inflammatory cells infiltrating the heart of exercised *mdx* mice by cytofluorimetric analysis. We found that total CD45+ hematopoietic cells were increased in the heart of exercised *mdx* mice compared to controls (Figure 5A,B). Within this cell population, the number of all immune cell subpopulations analyzed, including CD11b+ myeloid cells, CD11b+/F4/80^hi^ macrophages (MP), CD11b+F4/80+/Ly6c^hi^ recently recruited Mo/MP, CD11b+Ly6g+ neutrophils, and CD3+ T cells, increased in the exercised *mdx* heart compared to the aged-matched controls (Figure 5C). The level of immune cell infiltration found in the exercised *mdx* hearts was similar to the level observed in 11-month-old *mdx* (Supplementary Figure S5). Taken together, these results demonstrate that the exercise protocol hastens immune cell infiltration in the *mdx* heart, worsening the histo-pathological phenotype.

### 3.5. The Exercise Protocol Alters Mdx Heart Function

**Figure 6:**
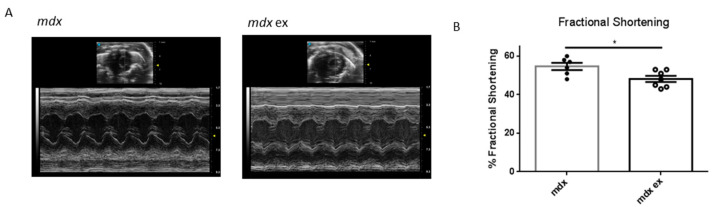
Echocardiographic analysis of left ventricle fractional shortening in exercised and control *mdx*: (**A**) representative images of the echocardiographic analysis of exercised and control *mdx* hearts, as indicated. (**B**) Left ventricle fractional shortening expressed as percentage and calculated on the short axe. Data are shown as mean ± S.E.M., *n* = 6/7 independent samples; * = *p* < 0.05, unpaired *t*-test w/Welch’s correction.

In DMD-related cardiomyopathy progression, fibrotic tissue deposition is associated with ventricular hypertrophy, and is followed by progressive left ventricle dysfunction, reduced heart contractility, and ejection volume. To determine whether the exercise protocol might lead to an alteration in heart function, we performed echocardiographic analysis in exercised and control *mdx* mice. There were no evident alterations in the dilation of the chambers, heart/body weight ratio, wall thickness, or heart rate (Table 1). However, as shown in Figure 6, the left ventricle fractional shortening in the heart of exercised *mdx* mice was significantly reduced compared with the age-matched controls. These results demonstrate that the increased cardiac fibrosis induced by exercise in *mdx* mice has a direct and measurable impact on heart function.

## 4. Discussion

This study demonstrates that long-term endurance exercise in *mdx* mice hastens the histo-pathological features of *mdx* dystrophic heart, in terms of fibrosis, inflammation, and function, making it a reliable model to test future therapeutic strategies. Indeed, one of the major stumbling blocks in developing such a strategy has been the absence of animal models mirroring the severity of the heart pathology seen in humans. The dystrophic pathology progression in the heart of the widely used model of DMD, the *mdx* mouse, is slower and milder in comparison to that of human patients [45].

As expected, we found that the extent of heart fibrosis in *mdx* mice increased with increasing age, and coincided with a peak of infiltrating immune cell, suggesting that fibrosis is driven by the inflammatory response. Interestingly, an earlier peak of immune cells’ infiltration was observed in the heart of 1-month-old *mdx* mice, at the age when skeletal muscle necrosis is very high [37,38]. Although not proven yet, we believe that this early peak depends on a systemic immune response, due to skeletal muscle damage, while the later one might represent a cardiac-specific response.

Although many papers investigated the effect of physical exercise on *mdx* skeletal muscle, only few of them assessed the impact of exercise on the heart. Physical exercise has been previously shown to activate p38 MAPK, JNK1, ERK1/2, or calcineurin signaling in *mdx* cardiac muscle [29], increase heart fibrosis [32], or impair heart function [34]. However, no detailed analysis of the inflammatory response and heart fibrosis has been conducted previously.

We found that our exercise protocol induced heart fibrosis in 3-mont-old *mdx* mice, at an age when no heart defects are detectable yet, and at a level comparable to that observed in 11-month-old mice. Interestingly, similar exercise protocols (i.e., chronic, moderate-intensity exercise) were described as beneficial for cardiac features in wild mice instead [46,47,48,49,50], suggesting that the effects we see in the heart of exercised *mdx* depend on altered susceptibility to work demand, due to a lack of dystrophin. Accordingly, cardiomyocyte necrosis and immune cells’ infiltration was observed in the heart of exercised *mdx* mice, also at levels comparable to those seen in 11-month-old *mdx* mice. These results suggest that endurance exercise leads to early cardiac fibrosis driven by necrosis-induced inflammatory response. Importantly, we show that the exercise-induced heart fibrosis impairs heart function, as the left ventricle fractional shortening significantly decreased in 3-month-old exercised *mdx* mice compared to controls. Indeed, the level of reduced fractional shortening is consistent with the level of fibrotic deposition observed [51]. Although it might appear mild, this decrease is meaningful in terms of prognosis, as it might be predictive of a late-onset and more pronounced left ventricular dysfunction.

To the best of our knowledge, this is the first study to characterize the histo-pathological phenotype of the heart in *mdx* mice upon exercise, analyzing all the key features of the dystrophic heart: immune cells infiltration, cardiomyocyte necrosis, ventricular fibrosis, and impaired function. We show that endurance, forced, and long-lasting exercise worsens the *mdx* heart phenotype, accelerating the progression of dystrophic cardiomyopathy without adversely affecting the diaphragm, and with only a moderate impact on limb muscle fibrosis. Although the level of heart damage is still not as severe as in the human disease, we believe that this protocol represents a fast, cheap, and easily reproducible way to worsen the *mdx* heart phenotype in order to evaluate the potential of novel therapeutic approaches aimed at ameliorating dystrophic heart pathology.

## Figures and Tables

**Table 1:** Summary of the main echocardiographic parameters in exercised and control *mdx*. Data are expressed as mean ± S.E.M., *n* = 6/7 independent samples. * = *p* < 0.05.

	End-Diastolic Diameter [mm]	End-Systolic Diameter [mm]	Anterior Wall Thickness [mm]	Posterior Wall Thickness [mm]	Fractional Shortening [%]	Heart Rate (bpm)	Heart/Body Weight [mg/g]
***mdx***	2.77 ± 0.07	1.27 ± 0.08	1.13 ± 0.04	1.08 ± 0.02	* 54.42 ± 1.89	452 ± 25.2	7.0 ± 0.24
***mdx* ex**	2.76 ± 0.06	1.44 ± 0.03	1.06 ± 0.04	1.11 ± 0.03	* 48.14 ± 1.61	443 ± 43.8	6.8 ± 0.17

## Data Availability

No new data were created or analyzed in this study. Data sharing is not applicable to this article.

## Supplementary Materials

The following are available online at https://www.mdpi.com/article/10.3390/life11070706/s1, Figure S1: Cytofluorimetric Gating Strategy; Figure S2: Quantification of immune cells populations in exercised and old mdx heart; Figure S3: Ventricular fibrosis in old and exercised mdx; Figure S4: Skeletal muscle is partially affected by the exercise protocol; Figure S5: Quantification of the immune population infiltrating exercised and control mdx mice.
