# Supplementary material for: Accelerating the Mdx Heart Histo-Pathology through Physical Exercise
Source: Life (Basel). 2021 Jul 17;11(7):706. doi: 10.3390/life11070706 (PMC8306456; doi:10.3390/life11070706)
Supplement: Supplementary file 1 [file life-11-00706-s001.zip › life-1274466-supplementary.pdf]

Accelerating the *mdx* heart histo-pathology through physical exercise

Jacopo Morroni[1], Leonardo Schirone[2], Daniele Vecchio[2], Carmine Nicoletti[1], Luca D'Ambrosio[2], Valentina Valenti[3], Sebastiano Sciarretta[2,4], Biliana Lozanoska-Ochser[1†], Marina Bouchè[1†*]

Supplementary Data

Supplementary Figure S1: Cytofluorimetric Gating Strategy

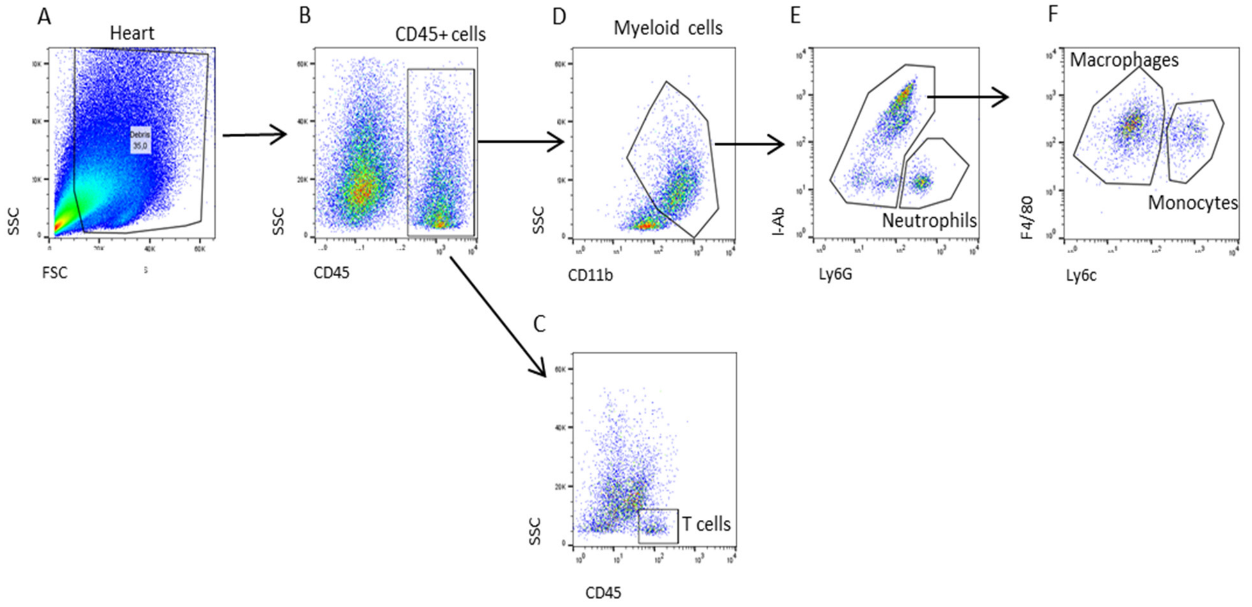

Representative images of our gating strategy for flow cytofluorimetric analysis. a) Heart infiltrating cells were isolated as described in Materials & Methods, and analysed with the instrument CyAn ADP (DAKO) and FlowJo software 10.1. b) Single, DAPI-negative (live) cells were then gated for their positivity to CD45 , to identify the hematopoietic lineage. c,d) CD45+ cells were then alternatively gated for their positivity to CD3 (T lymphocites) (c) or CD11b (myeloid population). e)Myeloid population was further divided in Ly6g+/I-Ab- cells (neutrophils) and Ly6g-/I-Ab+ cells. f) Ly6g-/I-Ab+ population was then split in F4/80+/Ly6c- cells (macrophages) and F4/80+/Ly6c^{hi} cells (freshly recruited monocytes).

Supplementary Figure S2: Immune cells infiltrate the *mdx* heart in two separate waves.

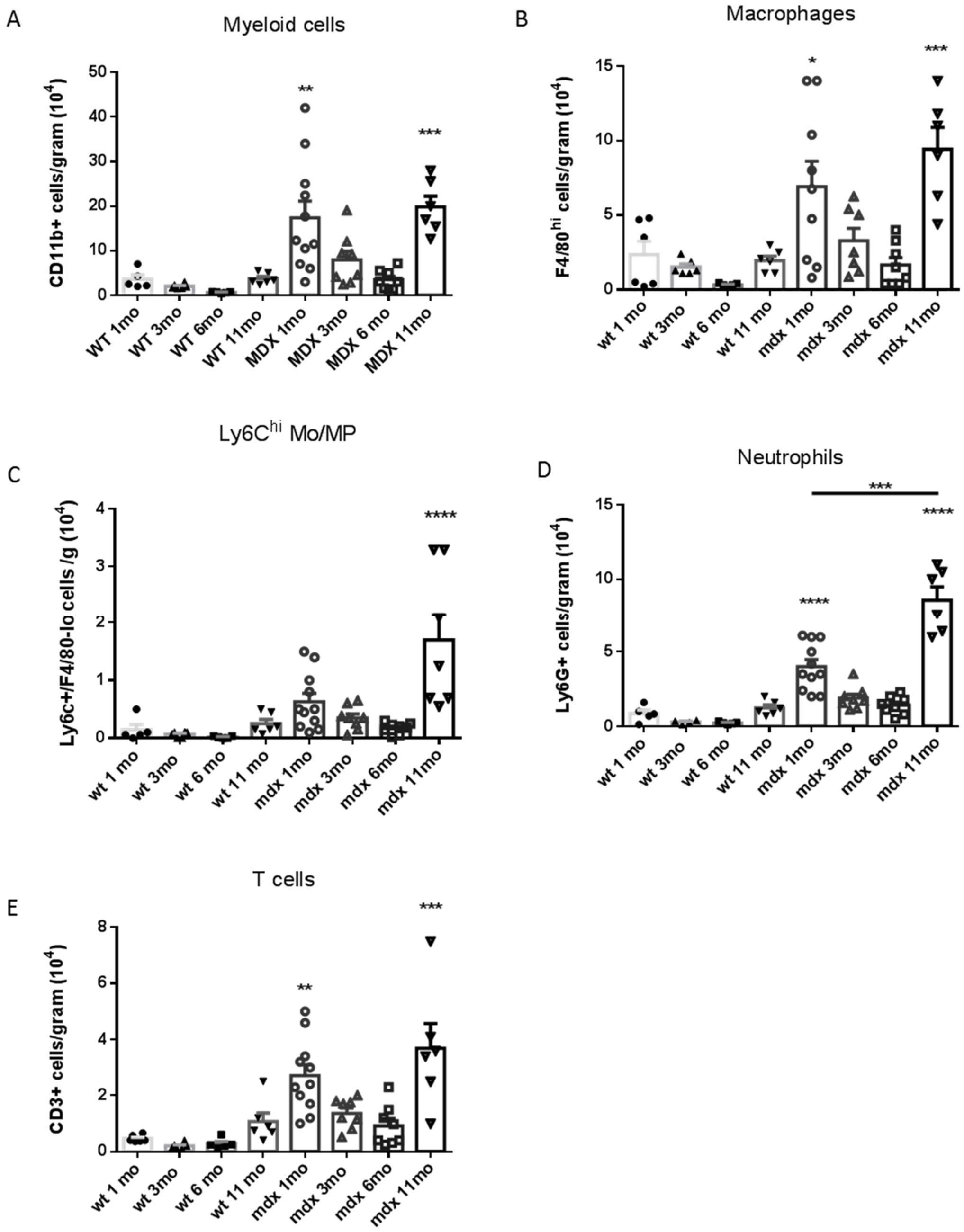

Quantification of immune cells populations in exercised and old *mdx* heart.

Quantification of the immune cells infiltrating wild-type or *mdx* heart at the indicated ages, expressed as number per gram of tissue. n= 5-11 independent samples. Data are shown as mean +/- S.E.M; *=p<0.05, **=p>0.01, ***=p<0.001, ****=p<0.0001, ordinary one-way ANOVA (Bonferroni correction for multiple comparisons). The cell populations identified are CD11b+ total myeloid cells (A), Ly6c-/F4/80^{hi} macrophages (B), Ly6c^{hi}/ F4/80- freshly recruited monocytes (C), Ly6g+ neutrophils (D) and CD3+ T cells (E)

Supplementary Figure S3: Old and exercised *mdx* display similar level of ventricular fibrosis.

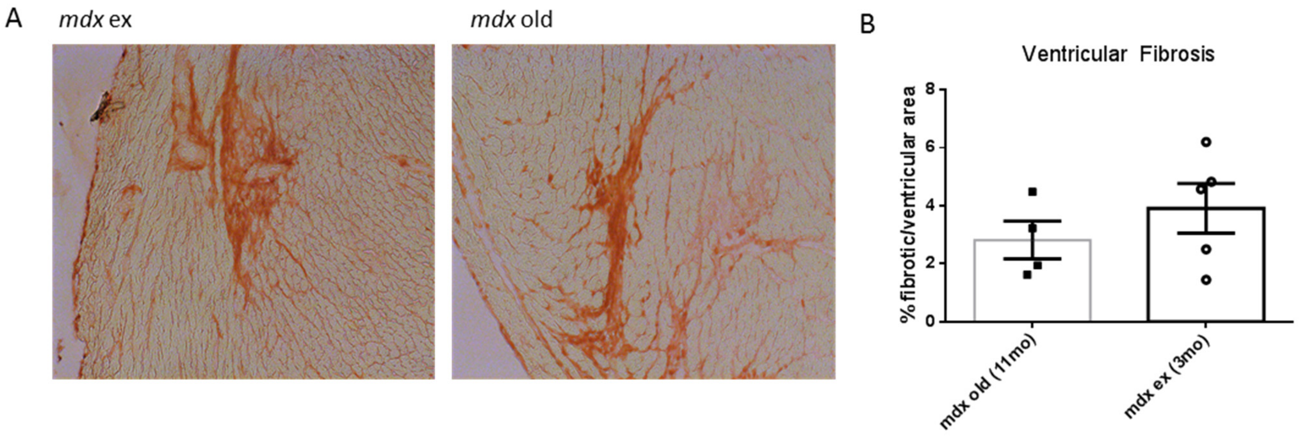

Ventricular fibrosis in old and exercised *mdx*. A) Representative images of Sirius red collagen staining of heart ventricles cryosections from 11mo-old and exercised *mdx* mice. Collagen deposition areas are indicated by the arrow. B) Quantification of heart ventricular fibrosis, determined by Sirius red collagen staining as in A. Data are expressed as percentage of ventricular fibrotic area over total ventricular area, as determined with ImageJ Colour Deconvolution plugin by G. Landini. n=4/5 independent samples.

Supplementary, Figure S4: Skeletal muscle is partially affected by the exercise protocol.

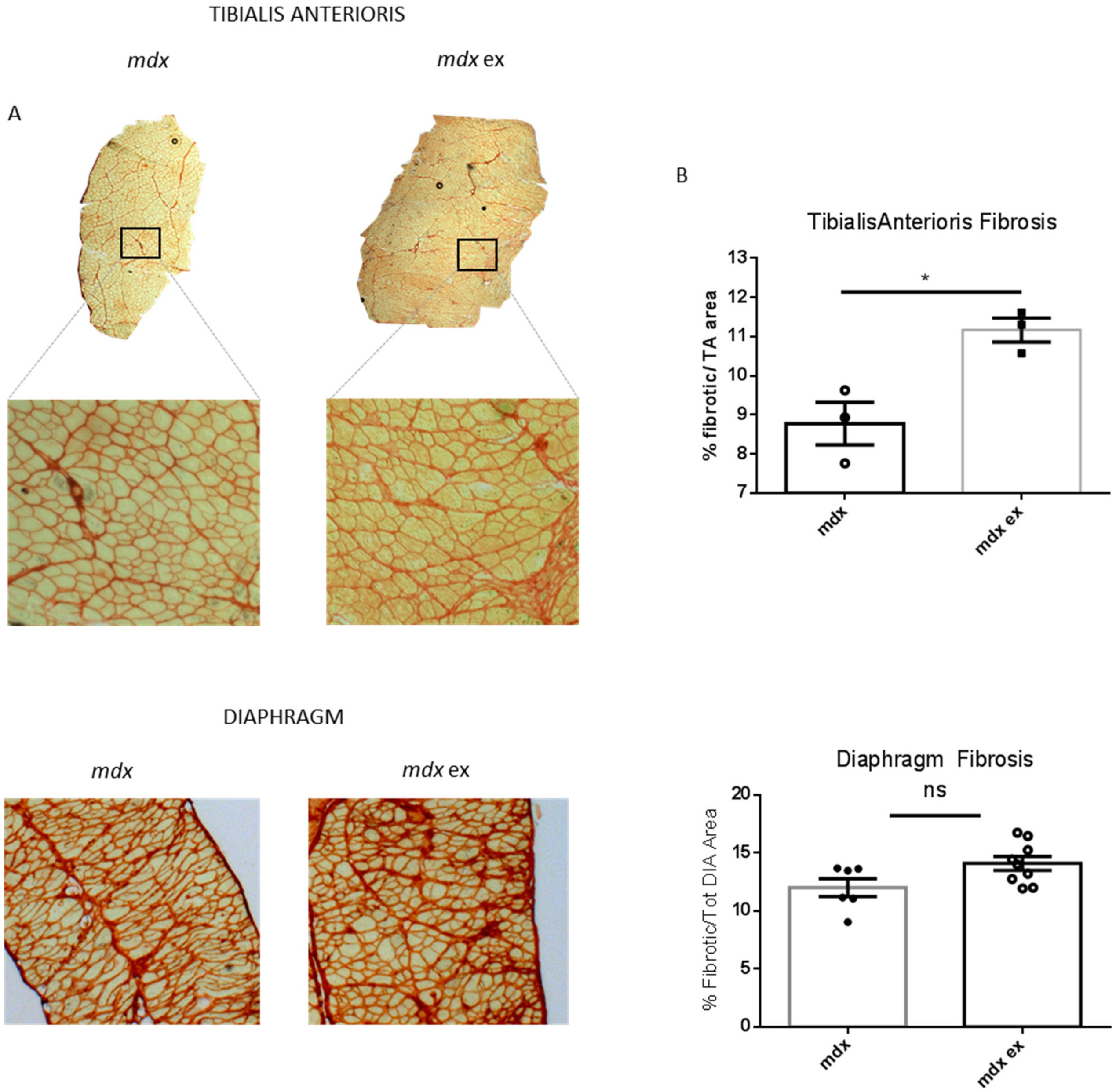

A) Representative images of Sirius red collagen staining of tibialis anterioris (TA) in exercised and control *mdx*. B) Quantification of TA fibrosis, determined by Sirius red collagen staining as in A. Data are expressed as percentage of fibrotic area over total TA area, as determined with ImageJ Colour Deconvolution plugin by G. Landini. n=3 independent samples. C) Representative images of Sirius red collagen staining of diaphragm (DIA) in exercised and control *mdx*. B) Quantification of DIA fibrosis, determined by Sirius red collagen staining as in A. Data are expressed as percentage of fibrotic area over total DIA area, as determined with ImageJ Colour Deconvolution plugin by G. Landini. n=6/9 independent samples

Supplementary Figure S5: Old and Exercised mdx mice display similar level of immune cell infiltration in the heart.

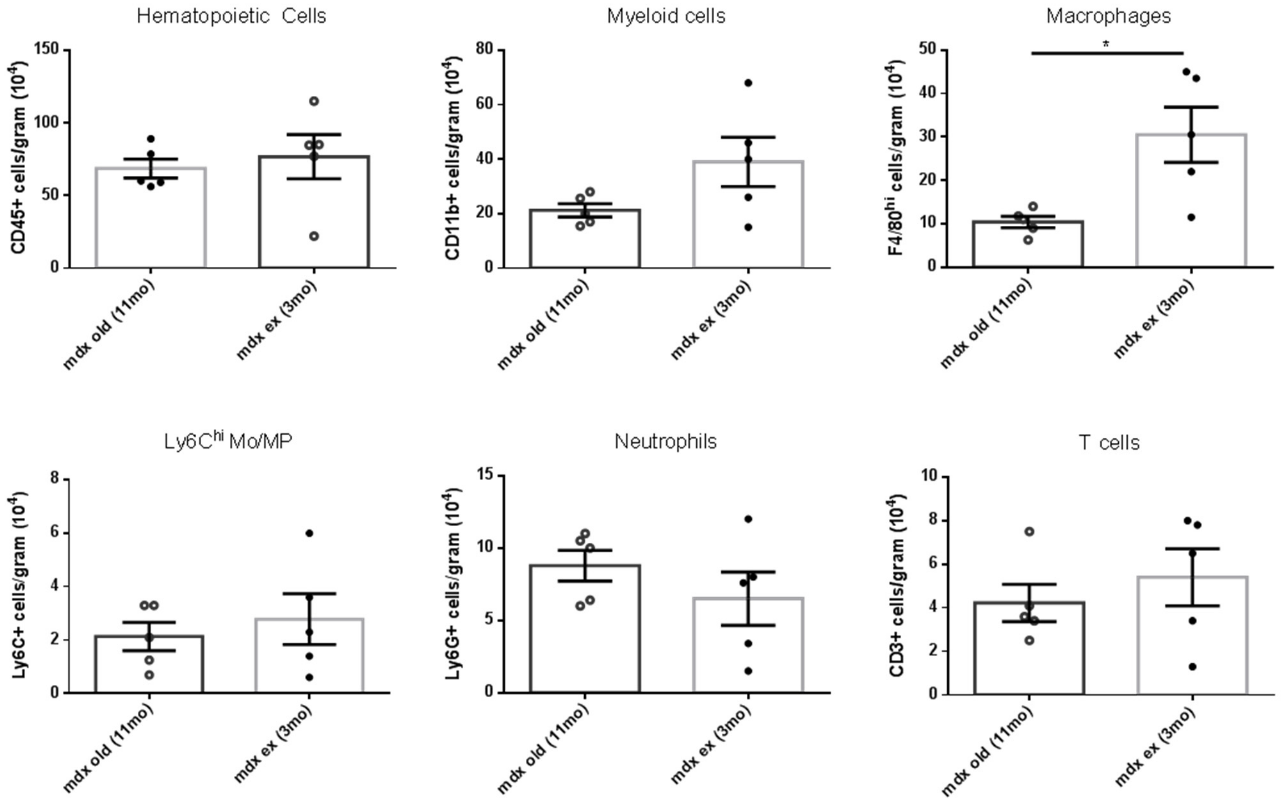

Quantification of the immune population infiltrating exercised and control *mdx* mice, expressed as number of cells normalized per gram of tissue. The cell populations identified are: CD45+ total hemopoietic cells (A), CD11b+ total myeloid cells (B), Ly6c-/F4/80^{hi} macrophages (C), Ly6c^{hi}/ F4/80- freshly recruited monocytes (D), Ly6g+ neutrophils (E) and CD3+ T cells (F). *=p>0.05, unpaired t-test w/Welch's correction.
